# Analysis of cases of acute occlusive abdomen in elderly patients

**Published:** 2015

**Authors:** OC Goidescu, T Patrascu

**Affiliations:** * Surgery Department, Municipal Hospital, Ramnicu Sarat, Romania; ** ”I. Juvara” Clinical Hospital, “Dr. I. Cantacuzino” Hospital, Bucharest, Romania

**Keywords:** acute abdomen, elderly patient, days delay, complications

## Abstract

Mechanisms of acute abdomen in the elderly patient are not different from the ones of young adults. What differs is the large number of associated diseases and specific geriatric pathology, ischemic disorders, diverticular disease. The diagnosis of acute abdomen in the elderly patient is difficult due to unclear symptoms, laboratory samples less modified and low cooperation. The article analyzed two groups of patients over 65 years, hospitalized in surgery in the last three years, with the diagnosis of acute surgical abdomen, from the point of view of the appearance of complications depending on the days of delay until surgery.

## Introduction

Mechanisms of acute abdomen in the elderly patient are not different from the ones of young adults. What differs is the large number of associated diseases and specific geriatric pathology, ischemic disorders, diverticular disease [**1**]. The diagnosis of acute abdomen in the elderly patient is difficult due to unclear symptoms, laboratory samples less modified and low cooperation [**2**]. The article analyzed two groups of patients over 65 years, hospitalized in surgery in the last three years, with the diagnosis of acute surgical abdomen, from the point of view of the appearance of complications depending on the days of delay until surgery.

Among the main causes of occlusion in the elderly patients, the following must be included: malignancies, volvulus, incisional and strangulated hernias, diverticular disease, postoperative adherent syndromes [**3**]. Even in this case, the diagnosis can be delayed; stopping the transit can initially be made because of habitual constipation. What is particular in these patients is the important pathological association often neglected delays in consulting a doctor and a poor anamnesis [**4**].

## Method

Two groups of patients with acute abdomen were analyzed before and after the introduction of working protocols [**5**]. The type of acute abdomen, associated diseases, the days of delay until surgery and postoperative complications were included in the analysis.

## Results

The first group included 60 patients. The age group I mainly consisted of Group 70 to 79 years and 80-89 years, groups 65-69 years and over 90 years were less represented.

**Fig. 1:**
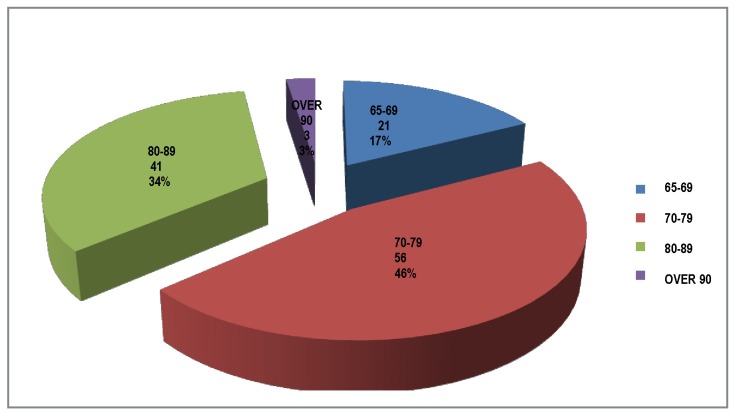
Age groups

For the 60 cases of occlusion, the causes were varied, but predominantly hernias and incisional hernias - 45 cases. The other 15 cases were represented by neoplasm 8 cases, volvulus 3 cases and accompanying occlusion in acute diseases 4 other cases.

**Fig. 2:**
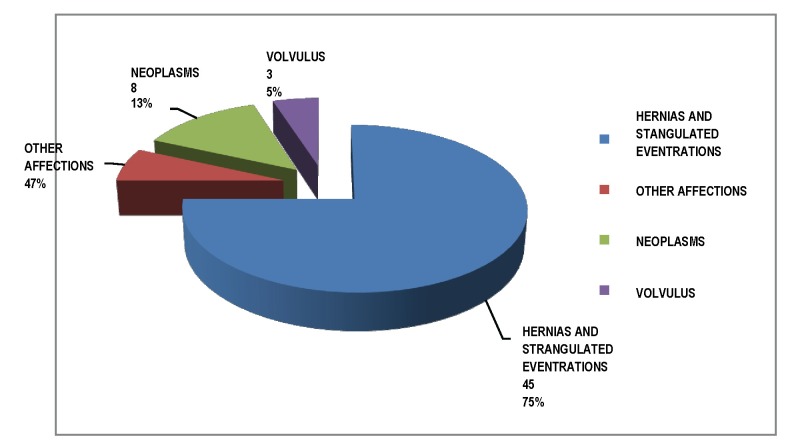
Occlusion causes

The age groups were dominated by the decade 80-89, followed by 70-79 years old. Nearly all the patients had at least one associated disease

**Table 1:** Data studied in Group I

Age groups	Occlusion	Cardiac	Renal	Diabetes	Other diseases	Days of delay	Complications
65-69	6	6	1	1	6	6	5
70-79	21	18	6	1	19	21	14
80-89	30	25	10	5	25	30	19
>90	3	3	3	1	3	3	1
Total	60	52	20	8	53	60	39

To improve the prognosis, the shortening of the time to surgery by introducing working protocols was tried.

The 2nd Group included 29 patients operated after the introduction of working protocols. The causes of the problems were similar, just like the pathological associations.

**Table 2:** Data studied in Group II

Age groups	Occlusions	Cardiac	Renal	Diabetes	Other diseases
65-69	2	2	1		2
70-79	12	11	2		12
80-89	15	14	7	4	15
Total	29	27	10	4	29

What was different in this group, were the days of delay and complications.

**Table 3:** The days of delay and complications in patients of Group II

**Days of delay**	**Patients**	**Complications**
1	19	9
2	4	4

At most 4 days of delay were found in this group, in 4 patients, all of them developing complications, while those with one day of delay also developed complications but in a smaller number, 9 to 19 patients.

The number of days of delay was proportional with the number of complications, 13 to 29 patients in the group which the protocol has been applied on, from 39 to 60 patients in the initial group.

**Fig. 3:**
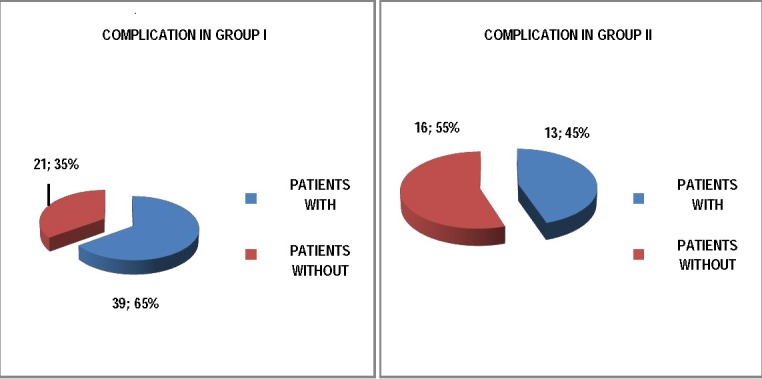
Proportion of complications in the two groups

## Discussion

The analysis of the two groups showed the decrease of the number of days of delay and complications after the introduction of working protocols. Postoperative complications that occurred in these patients were mostly due to the associated pathology but the establishment of an early diagnosis improved the prognosis. Therefore, the surgeon consulted any patient with abdominal complaints. These patients required a thorough medical history of both the current episode as well as in terms of chronic medication and personal pathologic history. The resuming of the questions during the anamnesis lasted for 2-3 hours or at request.

Direct investigations depending on the medical history and clinical examinations were made but with the imposition of a minimum set: X-ray for abdomen and chest, ultrasound, minimum of laboratory investigations. Patients required interdisciplinary consult according to the associated pathology.

## Conclusions

1. Regardless of the cause of acute abdomen and age, it is a serious disease.

2. The highest incidence is in the 7th and 8th decades.

3. The majority of patients have associated major diseases, most commonly cardiac, renal, diabetes, alcoholism, cerebral circulatory disorders.

4. Many patients have low adherence to treatment, the doctor of the guard being in front of the patients with known diseases hard but in therapeutic abandonment.

5. The clinical examination was poor in information.

6. An early diagnosis can improve the postoperative evolution.

**Disclosure:** none
